# Extrusion Combined with Fermentation Modifies Chemical Composition and Methionine Availability in Rapeseed Meal for Growing Pigs

**DOI:** 10.3390/ani16182835

**Published:** 2026-09-09

**Authors:** Aipeng Mao, Zexin Su, Jieying Jing, Junning Pu, Xiaohong Guo, Shufen Xue, Jing Fu, Jingyi Cai, Gang Jia, Gang Tian

**Affiliations:** Animal Nutrition and Efficient Feed Utilization Key Laboratory of Sichuan Province, Key Laboratory of Animal Disease-Resistance Nutrition of China Ministry of Education, Animal Nutrition Institute, Sichuan Agricultural University, Chengdu 611130, China; mao443418199@163.com (A.M.); 13689357792@163.com (Z.S.);

**Keywords:** digestibility, extrusion, fermentation, growing pig, rapeseed meal

## Abstract

Rapeseed meal (RSM) is a promising protein ingredient for animal feed; however, its utilization is limited by poor digestibility and the presence of anti-nutritional factors. In this study, we first investigated the effects of extrusion and fermentation on the structural characteristics and chemical composition of RSM. Subsequently, 18 growing pigs (6 pigs per treatment, 1 pig per replicate) were used. In period 1, pigs received diets with 20% RSM or fermented extruded rapeseed meal (FERSM) for nutrient digestibility determination. After a 7-day washout, the same pigs were re-assigned to 30% RSM or FERSM diets (6 pigs per treatment) for amino acid digestibility measurement. The results showed that extrusion combined with fermentation resulted in a more porous and loosened structure of RSM. Compared with RSM, FERSM exhibited a significant reduction in glucosinolate (GLs) content, whereas trichloroacetic acid-soluble protein (TCA-SP) content was significantly increased (*p_adj_* < 0.001). Furthermore, pigs fed the FERSM diet showed significantly higher standardized ileal digestibility (SID) and standardized ileal digestible content of methionine (Met) than those fed the RSM diet (*p_adj_* < 0.05). In conclusion, extrusion combined with fermentation, when wheat bran was included in the substrate, effectively modified the physicochemical properties and chemical composition of RSM and was associated with improved Met utilization in growing pigs.

## Simple Summary

Rapeseed meal is an important protein source for animal feed; however, its application is limited by poor digestibility and the presence of anti-nutritional factors, including glucosinolates, tannins, phytic acid, and dietary fiber. In this study, we found that extrusion combined with fermentation altered the structural characteristics of rapeseed meal and significantly changed the levels of trichloroacetic acid-soluble proteins and glucosinolates. Nutritional evaluation showed that the standardized ileal digestibility and standardized ileal digestible content of methionine was significantly improved in growing pigs fed fermented extruded rapeseed meal. These findings demonstrate that, when applied to RSM containing wheat bran, extrusion combined with fermentation is an effective strategy through which to improve the utilization of methionine.

## 1. Introduction

The increasing global demand for meat has accelerated the search for sustainable and cost-effective protein sources for livestock production. Rapeseed meal (RSM), the major by-product of rapeseed oil extraction, has attracted attention as a potential protein ingredient in animal feed due to its wide availability, low cost, relatively high protein content, and balanced amino acid (AA) profile [1,2]. However, the utilization of RSM in animal diets remains limited because of the presence of various anti-nutritional factors (ANFs), including glucosinolates (GLs), tannins, phytic acid, and dietary fiber [3]. Among these, GLs are considered the primary ANFs in RSM. High levels of GLs can negatively affect animal performance by reducing feed intake and impairing thyroid and liver functions [4]. Tannins may negatively affect nutrient utilization by precipitating proteins and inhibiting digestive enzymes, although low levels of specific tannins may have beneficial effects [5]. Phytic acid can reduce the availability of essential nutrients by binding to proteins, starch, lipids, and minerals, thereby impairing their utilization [6]. Therefore, reducing these unfavorable components is essential for improving the application of RSM in pig diets.

To enhance the nutritional value and utilization efficiency of RSM, various processing strategies, including heat treatment, microbial fermentation, and enzymatic hydrolysis, have been investigated in recent years [7,8,9]. Extrusion is an effective feed-processing technology that can reduce GLs content in RSM and improve the ileal digestibility of indispensable AA in growing pigs [10,11,12]. In addition, microbial fermentation has also been widely applied to improve the nutritional characteristics of RSM. Fermentation with *Saccharomyces cerevisiae* (*S. cerevisiae*) or *Saccharomyces boulardii* (*S. boulardii*) reduced the levels of GLs, isothiocyanates, polyphenolic compounds, carbohydrates, and organic acids, while increasing the content of some individual phenolic acids (gallic, *p*-coumaric, sinapic), crude protein and mineral [13]. Dietary supplementation with 10% fermented rapeseed meal (FRSM) produced by *S. cerevisiae* reduced the levels of inflammatory cytokines, including TNF-α and IL-1β in the colon and jejunum, as well as IL-8 and IL-6 in the ileum. Moreover, it decreased lipid peroxidation and thiobarbituric acid-reactive substance levels in plasma, jejunum, and colon [14]. *Aspergillus niger* (*A. niger*) is another microorganism widely used in solid-state fermentation because it secretes various extracellular degrading enzymes, particularly lignocellulose-degrading enzymes, acid proteases, and phytases. Solid-state fermentation with *A. niger* increased the contents of trichloroacetic acid-soluble protein (TCA-SP), crude protein (CP), small peptides, and ether extract (EE) in RSM while reducing the levels of ANFs, including neutral detergent fiber (NDF), GLs, isothiocyanates, oxazolythiothione, and phytic acid [15,16]. Dietary supplementation with 10% *A. niger*-fermented RSM improved average daily gain (ADG), feed conversion ratio (FCR), and calcium (Ca) and phosphorus (P) digestibility in growing pigs [17].

Furthermore, supplementation with 4–9% FRSM improved the digestibility of CP, EE, and crude fiber (CF) in sows, reduced diarrhea and mortality rates in piglets, and increased litter size and litter weight at 28 days of age [18]. Compared with pigs fed a rapeseed meal diet (RSMD), those fed a fermented rapeseed meal diet (FRSMD) exhibited higher apparent ileal digestibility (AID) of several amino acids such as His, Thr, Lys, and Ser. Additionally, digestible energy (DE), metabolizable energy (ME), and nitrogen utilization were improved in pigs fed FRSMD [19]. Fermentation of RSM has also been reported to enhance nutrient utilization and exert beneficial effects on meat quality and lipid metabolism in finishing pigs [20,21]. Therefore, microbial fermentation is an effective strategy for improving the nutritional value and utilization efficiency of RSM, while potentially reducing intestinal inflammation and enhancing antioxidant capacity in pigs.

Although fermentation and extrusion have individually demonstrated beneficial effects on improving the nutritional quality of RSM, the combined effects of these two processing technologies remain unclear. Therefore, the present study compared the structural characteristics and chemical composition of RSM and fermented extruded rapeseed meal (FERSM) and subsequently evaluated the utilization of nutrient and amino acid in growing pigs. The objective was to develop an effective processing strategy to improve the nutritional value of RSM and enhance its utilization as a protein source in pig diets.

## 2. Materials and Methods

### 2.1. Preparation of Fermented Extruded Rapeseed Meal

Rapeseed meal was ground and passed through a 40-mesh sieve before extrusion processing. The ground RSM was extruded using a DSE32 twin-screw extruder (Jinan Linyang Machinery Co., Ltd., Jinan, China) under the following conditions: moisture content of 15% (*w*/*w*), extrusion temperature of 145 °C, screw speed of 23 Hz, and feeding rate of 10 Hz. The extruded rapeseed meal was subsequently subjected to solid-state fermentation using a mixed microbial culture consisting of *Lactobacillus plantarum* (*L. plantarum*, CICC6053, China Center of Industrial Culture Collection, Beijing, China), *S. cerevisiae* (CICC 31564, China Center of Industrial Culture Collection, Beijing, China), and *A. niger* (CICC41258, China Center of Industrial Culture Collection, Beijing, China). The cell concentrations of *L. plantarum*, *S. cerevisiae*, and *A. niger* were adjusted to 2.33 × 10^8^ CFU/mL, 4.67 × 10^6^ CFU/mL, and ≥5 × 10^9^ CFU/mL, respectively. The three microorganisms were then mixed at a volumetric ratio of 2:1:3, and the mixed inoculum was added at a level of 9% (*v*/*w*) to the substrate. Wheat bran (15%, on DM basis of RSM) was added as an auxiliary fermentation substrate, and the solid-to-liquid ratio was adjusted to 1:1.2. Fermentation was conducted at 40 °C for 6 days under solid-state fermentation conditions. FERSM was produced in four independent fermentation batches, each using a separate lot of rapeseed meal as the substrate. After fermentation, the fresh samples were dried at 65 °C, ground in a Fitz mill, and stored at −20 °C. Subsequently, samples from each of the four batches were subjected to chemical analysis [22].

### 2.2. Comparison of Structural Characteristics and Chemical Composition Between Rapeseed Meal and Fermented Extruded Rapeseed Meal

#### 2.2.1. Scanning Electron Microscopy

Samples of RSM and FERSM were prepared for structural analysis. Briefly, a piece of conductive carbon tape was attached to an aluminum stub, and a small amount of sample powder was evenly dispersed onto the tape using a sample spoon. Loosely attached particles were removed by gently blowing with an ear bulb, leaving a stable layer of sample particles adhered to the tape surface. The prepared stubs were then placed into the chamber of a scanning electron microscope (SEM). The surface microstructures of RSM and FERSM were examined using a Sigma 360 field-emission scanning electron microscope (Carl Zeiss AG, Oberkochen, Germany) at an accelerating voltage of 3 kV. Images were captured at magnifications of 1000× and 5000×.

#### 2.2.2. Fourier Transform Infrared Spectroscopy

Fourier transform infrared spectroscopy (FTIR) was performed to analyze changes in the protein secondary structure of RSM and FERSM. Briefly, 2 mg of sample was thoroughly mixed with 200 mg of potassium bromide (KBr) and finely ground under an infrared lamp to ensure complete homogenization. The mixture was then compressed into a pellet using a mold. Pure KBr was used as the background reference. FTIR spectra were collected using an FTIR spectrometer (Nicolet iS10, Thermo Fisher Scientific Inc., Waltham, MA, USA) with 64 scans per sample. The obtained spectra were processed, and the relative proportions of protein secondary structures were determined by peak fitting using OMNIC and PeakFit v4.12 software. The amide I band (1600–1700 cm^−1^) was deconvoluted into different secondary structure components, with the characteristic peak regions assigned as follows: α-helix, 1650–1660 cm^−1^; β-sheet, 1600–1640 cm^−1^; β-turn, 1660–1700 cm^−1^; and random coil, 1640–1650 cm^−1^.

#### 2.2.3. Nutrient Composition

The contents of CP, EE, St (starch), CF, NDF, and acid detergent lignin (ADL) were analyzed according to the national standards of the People’s Republic of China (GB/T 6432–2018, GB/T 6433–2006, GB/T 42491–2023, GB/T 6434–2022, GB/T 20806–2022 and GB/T 20805–2006, respectively) [23,24,25,26,27,28]. The content of acid detergent fiber (ADF) was determined according to the agricultural industry standard of the People’s Republic of China (NY/T 1459–2022) [29].

#### 2.2.4. Glucosinolates

The GLs content was determined using a palladium chloride (PdCl_2_) colorimetric method according to the procedure described by [30]. Briefly, 0.1 g of sample was extracted with boiling distilled water in a water bath for 30 min. After cooling, the extract volume was adjusted to 10 mL and centrifuged at 5000 rpm for 10 min. Subsequently, 2 mL of the supernatant was mixed with 4 mL of 1 g/L sodium carboxymethyl cellulose (CMC-Na) solution and 2 mL of PdCl_2_ reagent. After incubation for 60 min, the absorbance (A_1_) was measured at 540 nm. To eliminate background interference, another 2 mL aliquot of the supernatant was mixed with CMC-Na solution and HCl (without PdCl_2_ reagent), and the absorbance (A_2_) was recorded after 120 min. The net absorbance value (A = A_1_ − A_2_) was calculated and used to determine GLs content based on a sinigrin standard curve.

#### 2.2.5. Trichloroacetic Acid-Soluble Protein

The content of trichloroacetic acid-soluble protein (TCA-SP) in the samples was determined according to the method described by Ovissipour et al. (2009) [31]. Briefly, freeze-dried RSM and FERSM samples (1.0 g) were suspended in 10 mL of distilled water (sample-to-solvent ratio 1:10, *w*/*v*) and extracted by magnetic stirring at 25 °C for 60 min. The suspension was centrifuged at 10,000× *g* for 15 min at 4 °C. The supernatant was collected and mixed with an equal volume of 30% (*w*/*v*) trichloroacetic acid (TCA) to achieve a final concentration of 15% (*w*/*v*) and then incubated at 4 °C for 30 min to precipitate large proteins. After centrifugation at 10,000× *g* for 15 min at 4 °C, the resulting supernatant was collected. The nitrogen content in the TCA-soluble supernatant was determined using the Kjeldahl method. The TCA-soluble protein/peptide content was calculated using a standard curve. All determinations were performed in quadruplicate (*n* = 4).

### 2.3. Comparison of Nutrient Digestibility Between Rapeseed Meal and Fermented Extruded Rapeseed Meal in Growing Pigs

#### 2.3.1. Experimental Design and Feeding Management

A total of 18 Duroc × Landrace × Yorkshire (DLY) castrated male pigs were housed individually in metabolic cages (2.5 m × 1.8 m × 0.8 m) with free water access and fed the experimental diets twice daily at 4% of body weight. Environmental conditions were kept at 20–25 °C and 60–80% relative humidity under natural light.

The nutrient digestibility trial consisted of two experimental stages. In the first stage, 18 pigs with an initial body weight of 25.62 ± 0.41 kg were randomly assigned to 3 dietary treatments, with 6 replicates per treatment and 1 pig per replicate. A corn–soybean meal diet (CSD) was formulated as the basal diet according to the nutrient requirements recommended by NRC (2012). The rapeseed meal diet I (RSMD I) and fermented extruded rapeseed meal diet I (FERSMD I) were formulated by including 20% RSM or FERSM in the basal diet, respectively [32]. The composition and nutrient levels of diets in the first phase are presented in Table S1. After a 6-day adaptation period, fecal samples were continuously collected for 4 consecutive days to determine nutrient digestibility. After a 7-day washout period was implemented, during which all pigs were fed a standard commercial diet, the same pigs (30.91 ± 0.59 kg) were re-allocated to 3 treatments, with 6 replicates per treatment and one pig per replicate. A nitrogen-free diet (NFD) based on corn starch was used to determine basal ileal endogenous nitrogen losses according to a previously described method [33]. The RSMD II and FERSMD II diets were formulated to contain 30% RSM or FERSM, respectively (Table S2), and all diets contained 0.4% chromium oxide (Cr_2_O_3_) as an indigestible marker. On day 7 after dietary adaptation, pigs were fed the experimental diets at 20-min intervals. Two hours after feeding, pigs were electrocuted and sequentially slaughtered to synchronize the digesta transit rate. Death was confirmed by the absence of pulse, respiratory movements, and corneal reflex prior to tissue collection. The abdominal cavity was opened, and the distal ileum was ligated. Digesta samples were collected from 20–25 cm proximal to the ileocecal valve and freeze-dried for analysis of DM, AA and Cr_2_O_3_ to calculate AID and SID.

#### 2.3.2. Determination of Nutrient Digestibility

The contents of gross energy (GE) in the samples were determined according to ISO 9831–1998 [34]. The contents of CP, EE, CF, NDF, ADF in samples were determined as described previously. The contents of DM, Ca, P, and AA were determined according to the following standards of the People’s Republic of China: GB/T 6435-2014, GB/T 6436–2018, GB/T 6437–2018, and GB/T 18246–2019 [35,36,37,38], respectively. The concentration of chromium oxide (Cr_2_O_3_) in diets and ileal digesta was determined using a colorimetric method according to the method described by Fenton, T.W. et al. (1979) [39].

The apparent total tract digestibility (ATTD) of nutrients was calculated using the following equation:

$$ATTD\left(\%\right)=\frac{Nutrient\;intake-Nutrient\;in\;feces}{Nutrient\;intake}\times 100$$

The digestibility of ingredient was calculated using the difference method as follows:

$$Di(\%)=\frac{Dm-\left(1-X\right)Dc}{X}\times 100$$

where Di is the digestibility of ingredient; Dm and Dc represent the digestibility coefficients of the mixed diet and the basal diet, respectively; and both were used in the formula as proportions. X is the proportion of the ingredient replacing the basal diet (0.20 in period 1 and 0.30 in period 2).

The apparent ileal digestibility (AID) of AA was calculated according to the method described by [33]:

$$AID(\%)=\left(1-\frac{{Marker}_{diet}}{{Marker}_{digesta}}\times \frac{{AA}_{digesta}}{{AA}_{diet}}\right)\times 100$$

The basal ileal endogenous amino acid losses (IAA_end_) were determined using pigs fed a nitrogen-free diet (NFD) according to the following equation:

$${IAA}_{end}={AA}_{digesta}\times \frac{{Marker}_{diet}}{{Marker}_{digesta}}$$

where IAA_end_ is the basal endogenous loss of AA, AA_digesta_ is the concentration of AA in the ileal digesta, and Marker_diet_ and Marker_digesta_ are the marker concentrations in diet and ileal digesta, respectively.

The standardized ileal digestibility (SID) of AA was calculated as follows:

$$SID\left(\%\right)=AID(\%)+\left(\frac{{IAA}_{end}}{{AA}_{intake}}\times 100\right)$$

where AA_intake_ represents the amino acid intake from the experimental diet.

The calculation of digestibility was performed individually for each pig, yielding six independent FERSM digestibility values per treatment (n = 6). The mean and SEM of these six values were then calculated for each treatment. All digestibility values were calculated on a dry-matter basis.

### 2.4. Statistical Analysis

All data are presented as mean ± standard error of the mean (SEM). Statistical analyses were conducted using SPSS 27.0 (SPSS Inc., Chicago, IL, USA). The homogeneity of variances was verified using Levene’s test, and comparisons between groups were conducted using the independent-samples Student’s *t*-test (or Welch’s *t*-test when variances were unequal). To account for the risk of Type I errors associated with multiple comparisons, all *p*-values were adjusted using Bonferroni correction. Statistical significance was set at *p_adj_* < 0.05 (*).

## 3. Results

### 3.1. Comparison of Structural Characteristics and Chemical Composition Between Rapeseed Meal and Fermented Extruded Rapeseed Meal

**Figure 1:**
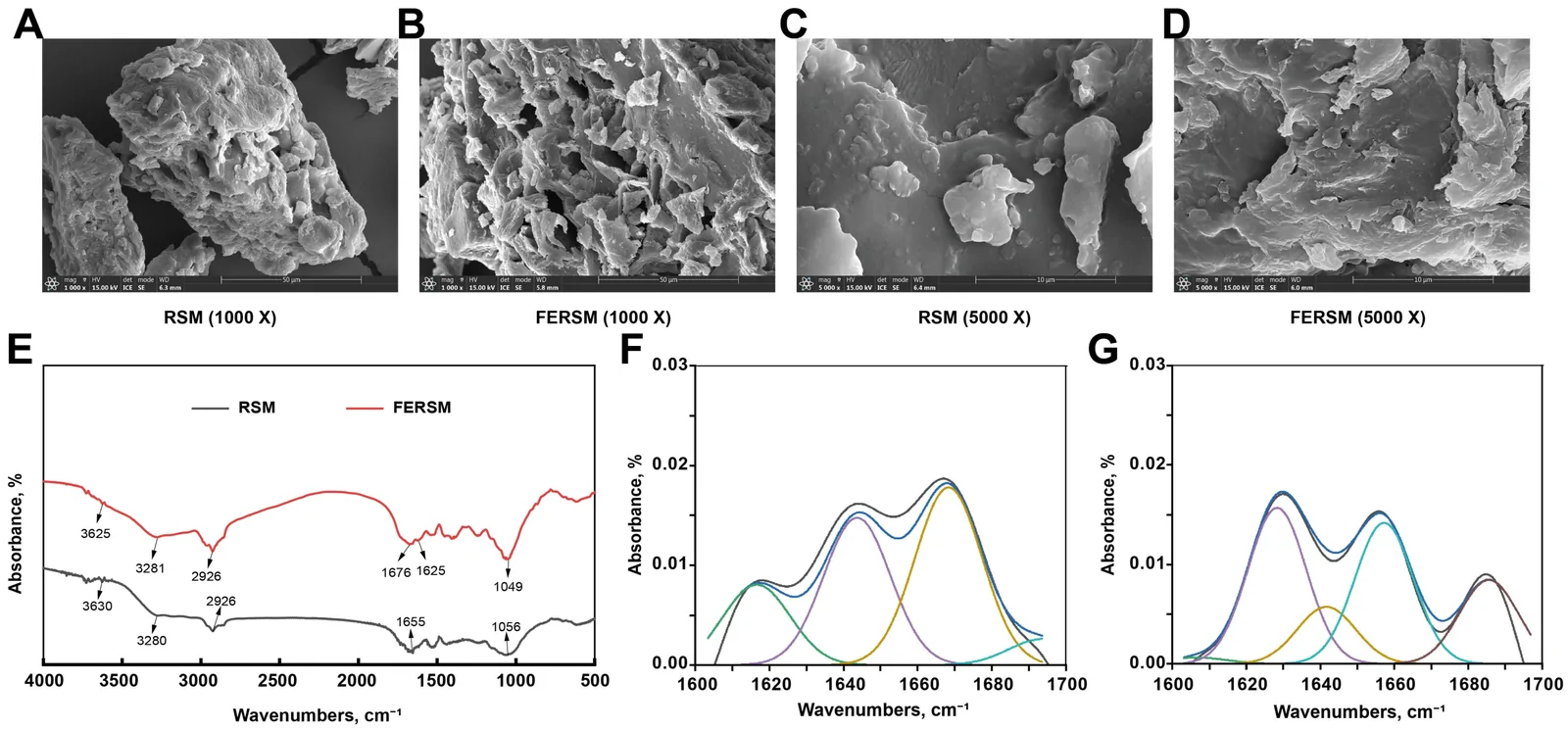
Comparison of structural characteristics between rapeseed meal and fermented extruded rapeseed meal. (**A**,**B**) Scanning electron micrographs of RSM and FERSM at 1000× magnification. (**C**,**D**) Scanning electron micrographs of RSM and FERSM at 5000× magnification. (**E**) FTIR spectra of RSM and FERSM. (**F**,**G**) Curve-fitted amide I region (1600–1700 cm^−1^) of the FTIR spectra of RSM and FERSM. RSM, rapeseed meal; FERSM, fermented extruded rapeseed meal; FTIR, Fourier transform infrared.

As shown in Figure 1, RSM exhibited a relatively smooth, compact, and well-organized surface structure. In contrast, FERSM displayed a markedly looser, more porous, and irregular morphology, suggesting substantial structural disruption induced by extrusion and fermentation. Compared with RSM, the characteristic peaks of FERSM at 3630 and 3280 cm^−1^ shifted slightly to 3625 and 3281 cm^−1^, respectively, indicating alterations in hydrogen bonding interactions and molecular environments after processing. In the amide region, two distinct peaks at 1676 and 1625 cm^−1^ appeared in the FTIR spectrum of FERSM, suggesting changes in protein secondary structures. For the polysaccharide-related absorption band, the peak maximum shifted from 1056 cm^−1^ in RSM to 1049 cm^−1^ in FERSM, accompanied by increased absorbance intensity, indicating an increase in polar functional groups after extrusion and fermentation.

The deconvolution profiles of the amide I region (1600–1700 cm^−1^) (Figure 1F,G) further demonstrated structural modifications of protein molecules. Extrusion combined with fermentation promoted partial unfolding of native proteins and altered intermolecular interactions among polypeptide chains.

### 3.2. Chemical Composition of Rapeseed Meal and Fermented Extruded Rapeseed Meal

As shown in Table 1, compared with RSM, the GLs content was decreased significantly in FERSM (*p**_adj_*** < 0.001), whereas the content of TCA-SP increased significantly in FERSM (*p**_adj_*** < 0.001). The contents of St and ADF were all reduced significantly in FERSM (*p**_adj_*** ≤ 0.001). Moreover, the concentrations of Arg, Lys, Phe and Thr were all reduced significantly in FERSM (*p**_adj_*** ≤ 0.001), and the concentrations of Asp, Ser, Glu, and Ala were also reduced significantly in FERSM (*p**_adj_*** < 0.01) (Table 2).

### 3.3. Nutrient and Amino Acid Digestibility of Rapeseed Meal and Fermented Extruded Rapeseed Meal in Growing Pigs

**Figure 2:**
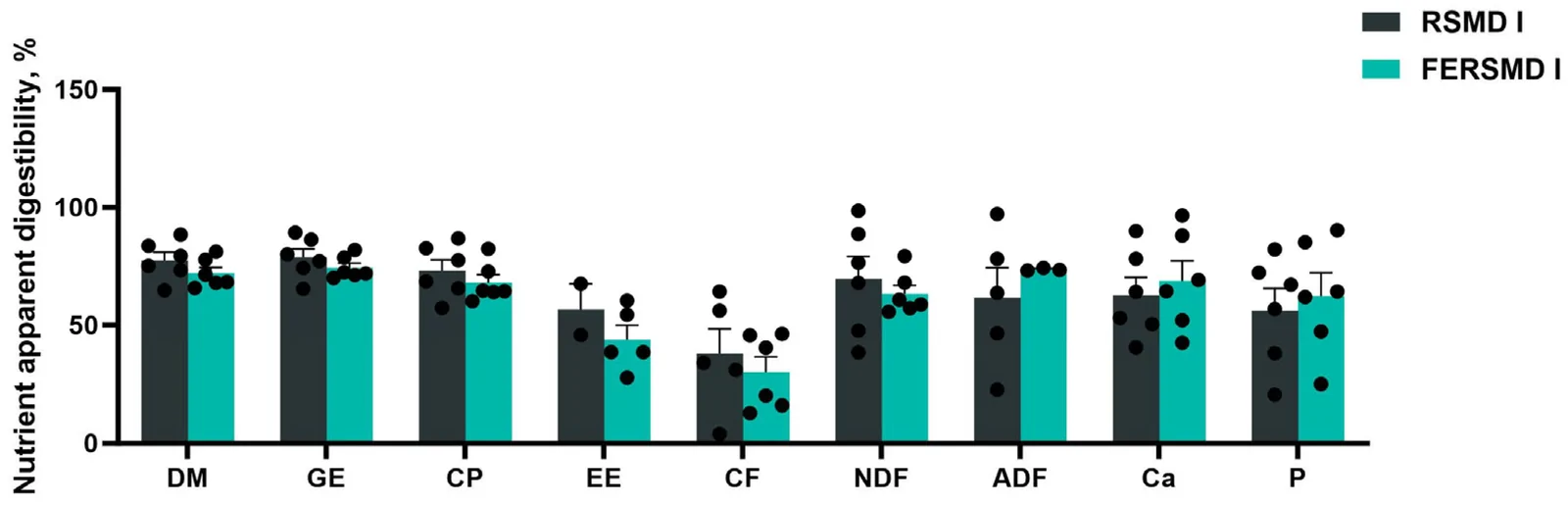
Comparison of nutrient digestibility between rapeseed meal and fermented extruded rapeseed meal in growing pigs. RSMD I, rapeseed meal diet I; FERSMD II, fermented extruded rapeseed meal diet II; DM, dry matter; GE, gross energy; CP, crude protein; EE, ether extract; CF, crude fiber; NDF, neutral detergent fiber; ADF, acid detergent fiber; Ca, calcium; P, phosphorus.

**Figure 3:**
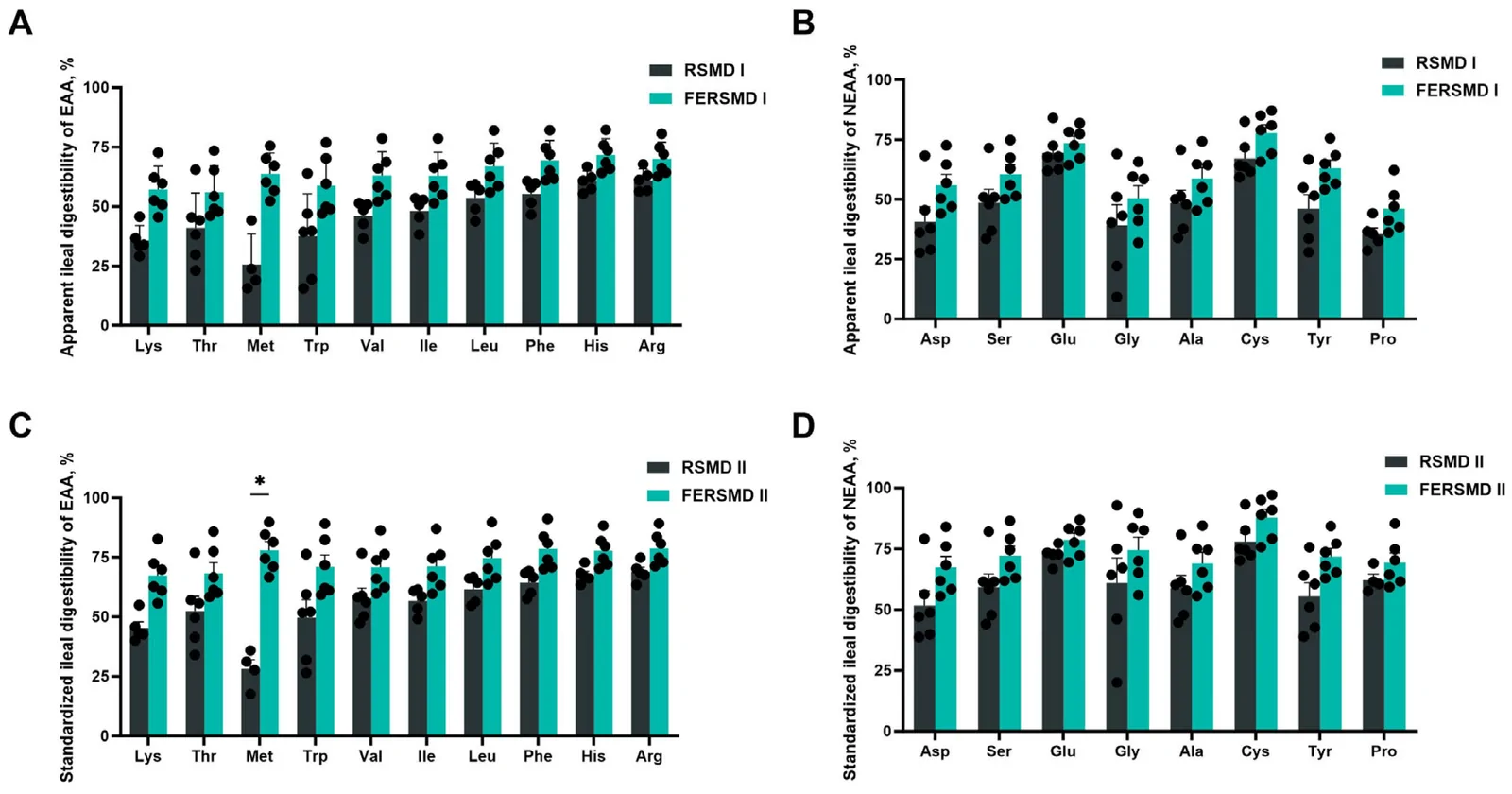
Comparison of amino acid digestibility between rapeseed meal and fermented extruded rapeseed meal in growing pigs. (**A**) AID of EAA. (**B**) AID of NEAA. (**C**) SID of EAA. (**D**) SID of NEAA. RSMD I, rapeseed meal diet I; FERSMD II, fermented extruded rapeseed meal diet II; AID, apparent ileal digestibility; SID, standardized ileal digestibility; EAA, essential amino acids; NEAA, non-essential amino acids.

As shown in Figure 2, compared with RSM, FERSM showed no significant differences in the apparent digestibility of DM, GE, CP, EE, CF, NDF, ADF, Ca, and P in growing pigs (*p_adj_* > 0.05). However, the SID of Met was significantly increased in pigs fed FERSM compared with those fed RSM (*p_adj_* < 0.05). The AID and SID of the other AAs were not significantly affected by FERSM supplementation (*p_adj_* > 0.05) (Figure 3).

### 3.4. Comparison of Apparent and Standardized Ileal Digestible Amino Acid Contents Between Rapeseed Meal and Fermented Extruded Rapeseed Meal

As presented in Table 3 and Table 4, the content of standardized ileal digestible Met was significantly higher in FERSM than in RSM (*p_adj_* < 0.01). while the apparent and standardized ileal digestible contents of all other amino acids did not differ significantly between two groups (*p_adj_* > 0.05).

## 4. Discussion

RSM has great potential as an alternative protein resource and feedstock for industrial biotechnology due to its abundant availability and nutritional value [40]. Biological transformation of RSM using microorganisms generally recognized as safe has been reported to reduce undesirable components, including GLs, tannins, erucic acid, and phytic acid, while improving its nutritional properties [41,42]. In the present study, extrusion combined with fermentation altered the surface morphology of RSM, resulting in a looser, more porous, and irregular structure. FTIR is an effective technique for analyzing chemical bonds and molecular structural changes [43] and has been widely applied to investigate modifications in protein and carbohydrate structures of feed ingredients [44,45,46]. Our results suggest that extrusion and fermentation altered the hydrogen bonding and glycosidic bond environments of polysaccharides and promoted conformational changes in protein structures, indicating substantial modifications in the molecular organization of RSM.

Further chemical analysis revealed that the GLs content decreased, whereas the TCA-SP proportion increased significantly after extrusion and fermentation. The increase in TCA-SP suggests that the combined processing promoted protein hydrolysis, converting macromolecular proteins into smaller peptides and free AAs. Previous studies have demonstrated that fermentation of hot-pressed RSM reduced 41.47% of NDF and 77.30% of GLs [47]. GLs can be hydrolyzed into goitrogens and other potentially toxic compounds, which may induce iodine deficiency and enlargement of the liver, kidney, and thyroid in animals; excessive GLs intake has also been associated with increased mortality [4]. However, low-GLs RSM can be safely incorporated into pig diets without adverse effects on growth performance or carcass traits [48]. Therefore, the reduction in GLs and increase in TCA-SP observed in the present study indicate that extrusion combined with fermentation effectively improved the chemical characteristics of RSM, enhancing its suitability as a protein ingredient in pig diets.

The T-cannulated pig model is the standard method for determining ileal amino acid digestibility. However, due to the unavailability of cannulated pigs in our facility, we selected the slaughter method for digesta collection, which has been validated as a viable alternative to T-cannulation in several published studies [49,50]. Previous studies have shown that fermentation can improve the nutritional value and sensory properties of RSM [51] and enhance the digestibility of protein and minerals in piglets [52,53]. However, in the present study, the contents of St, ADF and some amino acids (Arg, Lys, Phe, Thr, Asp, Ser, Glu, Ala) were reduced significantly in FERSM. These differences may be attributed to nutrient losses during extrusion processing and the metabolic activities of microorganisms during fermentation. The increase in TCA-SP and decrease in some amino acids indicate that extrusion and fermentation extensively hydrolyzed proteins into small peptides, while the liberated free amino acids were largely metabolized by microorganisms or lost via extrusion-induced chemical modifications [54,55]. Despite the reduction in nutrient concentrations, FERSM significantly improved the SID of Met, and the standardized ileal digestible content of Met was also increased in FERSM. This outcome may be attributable to the combined extrusion and fermentation process, which destroyed the cell wall matrix of RSM, degraded ANFs, and broke down macromolecular proteins into small peptides or free amino acids. These modifications increased the surface area for enzymatic hydrolysis and enhanced absorption efficiency in the terminal ileum. Met is generally regarded as the second or third limiting amino acid in practical swine diets. Dietary Met deficiency can impair growth performance, whereas excessive Met supplementation may cause adverse effects [56]. Met serves as a precursor for several functional metabolites, including S-adenosylmethionine (SAM) and homocysteine (Hcy), and appropriate Met or SAM supplementation has been reported to improve reproductive performance, intestinal morphology, hepatic lipid metabolism, muscle growth, and meat quality [56]. In similar studies with growing pigs, extrusion treatments at different screw speeds were also observed to significantly increase the AID and SID of Met [12]. Moreover, *A. niger*-fermented RSM significantly improved the AID of Lys and Met in growing pigs [57]. These findings are consistent with our results, further indicating that both extrusion and microbial fermentation can effectively enhance the utilization of Met in RSM. However, it should be noted that wheat bran was included in the fermentation substrate, and thus the observed effects cannot be attributed exclusively to the processing treatments.

## 5. Conclusions

In summary, extrusion combined with fermentation when wheat bran was included in the substrate effectively altered the physical structure and chemical composition of RSM and increased the SID and standardized ileal digestible content of Met in RSM. However, these processing treatments also led to reductions in certain nutrients and amino acids, whereas the apparent digestibility of nutrients was not significantly affected, which should be considered when optimizing the application of FERSM in pig diets.

## Figures and Tables

**Table 1:** Comparison of nutrient levels between rapeseed meal and fermented extruded rapeseed meal (DM basis, %).

Items	RSM	FERSM	*p*-Value	*p_adj_*-Value
GLs, μmol/g	60.32 ± 0.67	12.64 ± 0.27	<0.001	<0.001
TCA-SP	2.39 ± 0.06	9.11 ± 0.07	<0.001	<0.001
CP	40.49 ± 0.33	38.37 ± 0.31	0.006	0.648
EE	3.51 ± 0.10	2.57 ± 0.20	0.039	1
St	10.53 ± 0.17	3.44 ± 0.10	<0.001	<0.001
CF	13.49 ± 0.24	12.91 ± 0.32	0.239	1
NDF	38.36 ± 0.78	38.04 ± 0.22	0.750	1
ADF	27.67 ± 0.81	16.02 ± 0.88	<0.001	0.001
ADL	19.34 ± 0.82	16.76 ± 0.56	0.049	1

RSM, rapeseed meal; FERSM, fermented extruded rapeseed meal. GLs, glucosinolates; TCA-SP, trichloroacetic acid-soluble protein; CP, crude protein; EE, ether extract; St, starch; CF, crude fiber; NDF, neutral detergent fiber; ADF, acid detergent fiber; ADL, acid detergent lignin; Ash, crude ash.

**Table 2:** Comparison of AA levels between rapeseed meal and fermented extruded rapeseed meal (DM basis, %).

Items	RSM	FERSM	*p*-Value	*p_adj_*-Value
EAA				
Arg	2.16 ± 0.01	1.56 ± 0.02	<0.001	<0.001
His	1.25 ± 0.01	1.09 ± 0.01	<0.001	0.245
Ile	1.51 ± 0.01	1.48 ± 0.01	0.055	1
Leu	2.54 ± 0.01	2.44 ± 0.01	0.001	0.108
Lys	2.21 ± 0.01	1.55 ± 0.02	<0.001	<0.001
Met	0.68 ± 0.04	0.69 ± 0.01	0.640	1
Phe	1.51 ± 0.00	1.40 ± 0.01	<0.001	<0.001
Thr	1.73 ± 0.01	1.34 ± 0.00	<0.001	0.001
Trp	0.50 ± 0.01	0.44 ± 0.01	0.005	0.54
Val	2.08 ± 0.01	2.04 ± 0.08	0.624	1
NEAA				
Asp	2.67 ± 0.01	2.44 ± 0.02	<0.001	0.002
Ser	1.55 ± 0.01	1.00 ± 0.01	<0.001	<0.001
Glu	7.10 ± 0.03	6.26 ± 0.08	<0.001	0.006
Gly	1.97 ± 0.01	1.91 ± 0.02	0.011	1
Ala	1.72 ± 0.01	1.53 ± 0.01	<0.001	0.004
Cys	0.38 ± 0.01	0.41 ± 0.04	0.486	1
Tyr	0.60 ± 0.01	0.53 ± 0.05	0.226	1
Pro	3.29 ± 0.03	2.76 ± 0.07	0.003	0.324

RSM, rapeseed meal; FERSM, fermented extruded rapeseed meal; EAA, essential amino acids; NEAA, non-essential amino acids.

**Table 3:** Comparison of apparent ileal digestible amino acid contents between RSM and FERSM (g/kg DM).

Items	RSM	FERSM	*p*-Value	*p_adj_*-Value
EAA				
Arg	13.17 ± 0.46	10.93 ± 0.44	0.007	0.756
His	7.55 ± 0.25	7.85 ± 0.30	0.477	1
Ile	7.29 ± 0.43	9.28 ± 0.60	0.029	1
Leu	13.60 ± 0.78	16.33 ± 0.97	0.063	1
Lys	7.93 ± 0.61	8.87 ± 0.61	0.305	1
Met	1.76 ± 0.44	4.36 ± 0.25	<0.001	0.052
Phe	8.34 ± 0.41	9.71 ± 0.47	0.062	1
Thr	7.12 ± 1.04	7.52 ± 0.60	0.744	1
Trp	1.87 ± 0.37	2.56 ± 0.22	0.139	1
Val	9.57 ± 0.58	12.85 ± 0.83	0.013	1
NEAA				
Asp	10.89 ± 1.63	13.66 ± 1.10	0.189	1
Ser	7.51 ± 0.85	6.08 ± 0.40	0.156	1
Glu	49.31 ± 2.36	46.06 ± 1.74	0.294	1
Gly	7.72 ± 1.70	9.63 ± 1.01	0.358	1
Ala	8.34 ± 0.91	9.00 ± 0.68	0.577	1
Cys	2.55 ± 0.13	3.19 ± 0.14	0.009	0.972
Tyr	2.76 ± 0.35	3.35 ± 0.17	0.165	1
Pro	11.70 ± 0.84	12.72 ± 1.10	0.495	1

RSM, rapeseed meal; FERSM, fermented extruded rapeseed meal; EAA, essential amino acids; NEAA, non-essential amino acids.

**Table 4:** Comparison of standardized ileal digestible amino acid contents between RSM and FERSM (g/kg DM).

Items	RSM	FERSM	*p*-Value	
EAA				
Arg	14.88 ± 0.40	12.28 ± 0.44	0.002	0.216
His	8.43 ± 0.19	8.51 ± 0.30	0.822	1
Ile	8.58 ± 0.36	10.52 ± 0.60	0.028	1
Leu	15.65 ± 0.65	18.21 ± 0.97	0.065	1
Lys	10.03 ± 0.57	10.46 ± 0.61	0.629	1
Met	1.92 ± 0.27	5.35 ± 0.25	<0.001	0.002
Phe	9.70 ± 0.35	10.98 ± 0.47	0.068	1
Thr	9.10 ± 1.05	9.17 ± 0.60	0.953	1
Trp	2.48 ± 0.37	3.09 ± 0.22	0.191	1
Val	11.28 ± 0.47	11.42 ± 0.83	0.012	1
NEAA				
Asp	13.80 ± 1.63	16.45 ± 1.10	0.207	1
Ser	9.15 ± 0.84	7.25 ± 0.40	0.069	1
Glu	51.54 ± 1.20	49.21 ± 1.74	0.318	1
Gly	12.02 ± 2.03	14.23 ± 1.01	0.353	1
Ala	10.09 ± 0.89	10.57 ± 0.68	0.681	1
Cys	2.96 ± 0.13	3.60 ± 0.14	0.009	0.972
Tyr	3.32 ± 0.34	3.81 ± 0.17	0.226	1
Pro	20.47 ± 0.81	19.12 ± 1.10	0.400	1

RSM, rapeseed meal; FERSM, fermented extruded rapeseed meal; EAA, essential amino acids; NEAA, non-essential amino acids.

## Data Availability

The data that support the findings of this study are available from the corresponding author upon reasonable request.

## Supplementary Materials

The following supporting information can be downloaded at https://www.mdpi.com/article/10.3390/ani16182835/s1, Table S1: Composition and nutrient levels of diets in the first phase (as-fed basis, %); Table S2: Composition and nutrient levels of diets in the second phase (as-fed basis, %).

## Abbreviations

The following abbreviations are used in this manuscript:

*A. niger*	*Aspergillus niger*
AA	amino acid
ADF	acid detergent fiber
ADG	average daily gain
AID	apparent ileal digestibility
ANFs	anti-nutritional factors
Ash	crude ash
Ca	calcium
CF	crude fiber
CP	crude protein
CSD	corn–soybean meal diet
DM	dry matter
EAA	essential amino acids
EE	ether extract
FCR	feed conversion ratio
FERSM	fermented extruded rapeseed meal
FERSMD	fermented extruded rapeseed meal diet
FRSM	fermented rapeseed meal
FTIR	Fourier transform infrared spectroscopy
GE	gross energy
GLs	glucosinolates
*L. plantarum*	*Lactobacillus plantarum*
NDF	neutral detergent fiber
NEAA	non-essential amino acids
NFD	nitrogen-free diet
P	phosphorus
RSM	rapeseed meal
RSMD	rapeseed meal diet
*S. boulardii*	*Saccharomyces boulardii*
*S. cerevisiae*	*Saccharomyces cerevisiae*
SEM	scanning electron microscope
SID	standardized ileal digestibility
St	starch
TCA-SP	trichloroacetic acid-soluble protein
