# Supplementary material for: Extrusion Combined with Fermentation Modifies Chemical Composition and Methionine Availability in Rapeseed Meal for Growing Pigs
Source: Animals (Basel). 2026 Sep 9;16(18):2835. doi: 10.3390/ani16182835 (PMC13603813; doi:10.3390/ani16182835)
Supplement: Supplementary file 1 [file animals-16-02835-s001.zip › animals-4521655-supplementary.pdf]

Table S1 Composition and nutrient levels of diets in the first phase (as-fed basis, %)

Ingredients	CSD	RSMD I	FERSMD I
Corn	74.27	59.42	59.42
Soybean meal	23.00	18.40	18.40
RSM	0.00	19.30	0.00
FERSM	0.00	0.00	19.42
$CaCO_3$	0.25	0.40	0.28
$CaHPO_4$	1.50	1.50	1.50
NaCl	0.50	0.50	0.50
Choline chloride	0.15	0.15	0.15
Vitamin premix[1)]	0.03	0.03	0.03
Mineral premix[2)]	0.30	0.30	0.30
Total	100.00	100.00	100.00
Nutrients levels[3)]			
DM	87.22	87.28	87.29
GE/(MJ/kg)	16.39	16.39	16.39
DE/(MJ/kg)	13.91	13.55	13.64
CP	15.28	19.35	19.65

Note: 1) Vitamin premix provide per kg of diets: VA 9 000.0 IU, VB_1 3.0 mg, VB_2 7.5mg, VB_6 3.6mg, VB_{12} 0.36 mg, VD_3 3 000.0 IU, VK_3 3.0 mg, VE 24.0 IU, Niacin 30.0 mg, Folic acid 1.5 mg, Pantothenic acid 15.0 mg, Biotin 1.5 mg.

2) Mineral premix provided per kg of diets: Fe ($FeSO_4 \cdot H_2O$) 100.0 mg, Cu ($CuSO_4 \cdot 5H_2O$) 5.0 mg, Zn ($ZnSO_4 \cdot H_2O$) 80.0 mg, Mn ($MnSO_4 \cdot H_2O$) 3.0 mg, I (KI) 0.14 mg, Se (Na_2SeO_3) 0.25 mg.

3) In nutritional levels, GE was a calculated value, while the others were measured values.

4) CSD, corn and soybean meal diet; RSMD I, rapeseed meal diet I; FERSMD I, fermented extruded rapeseed meal diet I; RSM, rapeseed meal; FERSM, fermented extruded rapeseed meal.

Table S2 Composition and nutrient levels of diets in the second phase (as-fed basis, %)

Ingredients	NFD	RSMD II	FERSMD II
RSM	0.00	30.00	0.00
FERSM	0.00	0.00	30.00
Corn starch	80.54	54.54	54.54
Cellulose	4.00	0.00	0.00
Saccharose	10.00	10.00	10.00
Soybean oil	2.00	2.00	2.00
$CaCO_3$	0.18	0.18	0.18
$CaHPO_4$	1.90	1.90	1.90
NaCl	0.50	0.50	0.50
Choline chloride	0.15	0.15	0.15
Vitamin premix[1)]	0.03	0.03	0.03
Mineral premix[2)]	0.30	0.30	0.30
Cr_2O_3	0.40	0.40	0.40
Total	100.00	100.00	100.00
Nutrients levels[3)]			
DM	89.24	89.07	88.62
GE/(MJ/kg)	15.03	15.35	15.31
CP	0.61	11.26	11.48

Note: 1) Vitamin premixes provide per kg of diets: VA 9 000.0 IU, VB_1 3.0 mg, VB_2 7.5mg, VB_6 3.6mg, VB_{12} 0.36 mg, VD_3 3 000.0 IU, VK_3 3.0 mg, VE 24.0 IU, Niacin 30.0 mg, Folic acid 1.5 mg, Pantothenic acid 15.0 mg, Biotin 1.5 mg.

2) Mineral premixes provide per kg of diets: Fe ($FeSO_4 \cdot H_2O$) 100.0 mg, Cu ($CuSO_4 \cdot 5H_2O$) 5.0 mg, Zn ($ZnSO_4 \cdot H_2O$) 80.0 mg, Mn ($MnSO_4 \cdot H_2O$) 3.0 mg, I (KI) 0.14 mg, Se (Na_2SeO_3) 0.25 mg.

3) Nutrient levels were measured values.

4) NFD, nitrogen-free diet; RSMD II, rapeseed meal diet II; FERSMD II, fermented extruded rapeseed meal diet II; RSM, rapeseed meal; FERSM, fermented extruded rapeseed meal.
